# Carrier model of HTLV-1 infection in humanized NOG mice

**DOI:** 10.1186/1742-4690-11-S1-P42

**Published:** 2014-01-07

**Authors:** Kenta Tezuka, Mami Tei, Takaharu Ueno, Runze Xun, Jun-ichi Fujisawa

**Affiliations:** 1 Department of Microbiology, Kansai Medical University, Hirakata, Osaka, Japan

## 

HTLV-1 infection leads to adult T-cell leukemia (ATL) after long latent infection period. We have established an HTLV-1-infected humanized NOG mouse model to analyze the mechanism of ATL development. Humanized mice were inoculated with Jurkat-based HTLV-1 producing cells, JEX, into peritoneal cavity to infect human T-cells with HTLV-1. Infected T-cells proliferated vigorously in infected mice and the mice died of ALT-like lymphoproliferative disorder with attenuated HTLV-1 specific immunoresponses within two to three months of infection. In contrast, when HTLV-1 producing cells were inoculated orally, infection of human T-cells was established but the proviral loads (PVL) was kept constant in small amount (0.2-9.5 copies / 100 PBMCs) without any pathological feature, even after 18 weeks post infection. The amount of antibody against HTLV-1 in the plasma of orally infected mice was equivalent to that of intraperitoneally infected mice at 2-6 weeks post infection. The titer of antibody was maintained throughout lifetime in orally infected mice but not in intraperitoneally infected mice. Furthermore, depletion of CD8 T-cells from orally infected mice by injecting anti-CD8 antibody resulted in transient increase of PVL in PBMCs, indicating the involvement of cytotoxic T-cells in the control of PVL in these mice. Thus, orally infected humanized NOG mice should provide a model system of HTLV-1 carrier to examine factors involved in the development of ATL from carrier state and play a significant role in evaluating preventive vaccine candidates and antiretroviral drugs for HTLV-1 infection.

